# Epidemiology of sepsis in Brazil: Incidence, lethality, costs, and other indicators for Brazilian Unified Health System hospitalizations from 2006 to 2015

**DOI:** 10.1371/journal.pone.0195873

**Published:** 2018-04-13

**Authors:** Ricardo Alfredo Quintano Neira, Silvio Hamacher, André Miguel Japiassú

**Affiliations:** 1 Industrial Engineering Department, Pontifícia Universidade Católica do Rio de Janeiro, Rio de Janeiro, Rio de Janeiro, Brazil; 2 Philips Research Brazil, Barueri, São Paulo, Brazil; 3 Instituto Nacional de Infectologia, Fundação Oswaldo Cruz, Rio de Janeiro, Rio de Janeiro, Brazil; Universidade do Extremo Sul Catarinense, BRAZIL

## Abstract

**Background:**

Sepsis is considered a major worldwide health burden, with high mortality and associated costs. Health indicators are essential to define strategies to improve the treatment of diseases, and the epidemiology information of sepsis in developing countries is scarce. Thus, the aim of this work is to assess trends in the incidence, lethality, costs, and other indicators of sepsis for Brazilian Unified Health System (SUS—*Sistema Único de Saúde*) hospitalizations for the period from January 2006 to December 2015.

**Materials and methods:**

We conducted this study using data from the SUS hospital information system. We selected registries of SUS hospitalizations of patients diagnosed with sepsis (total of 724,458 cases from 4,271 public and private Brazilian hospitals).

**Results:**

From 2006 to 2015, the annual sepsis incidence increased 50.5% from 31.5/100,000 to 47.4/100,000 persons. The mean hospital length of stay (LOS) was 9.0 days. A total of 29.1% of the hospitalizations had admission to the intensive care unit (ICU) with a mean ICU LOS of 8.0 days. The mean cost per hospitalization was US$624.0 and for hospitalizations requiring intensive care was U$1,708.1. The overall sepsis lethality rate was 46.3%, and for hospitalizations with admission to the ICU, it was 64.5%. During the study period, the lethality rate for children/teenagers decreased 40.1%, but for all other age groups it increased 11.4%. The sepsis lethality rate in public hospitals (55.5%) was higher than private hospitals (37.0%) (p < 0.001). The mean hospitalization LOS for public hospitals (10.3 days) was higher than private hospitals (7.6 days) (p < 0.001).

**Conclusions:**

The incidence and lethality rate of sepsis increased in SUS hospitalizations during the study period. The SUS’s low reimbursement to hospitals for treating sepsis may be one of the reasons for the high lethality rate.

## Introduction

According to the Third International Consensus Definitions for Sepsis and Septic Shock (Sepsis-3), sepsis is defined as “life-threatening organ dysfunction caused by a dysregulated host response to infection" [1].

The incidence of sepsis is growing and is associated with high mortality rates, consisting of a healthcare and economic burden. In the United States, research studies [2–6] have shown an increase in sepsis incidence. Kumar et al. [3] present an incidence increase of 140% from 2000 to 2007, and Stoller et al. [4] present an annual incidence increase of 26% from 2008 to 2012. Health professionals and economic costs associated with sepsis are high [7,8]. Costs vary according to each country and study, and factors like age, the severity of sepsis, and type of institutions influence costs. For example, the geometric mean cost for sepsis in the United States was US$19,330 (2007)[6]; the median cost per episode of patients admitted in 21 Brazilian ICUs was US$9,632 (2003–2004) [9]; the mean cost per hospitalization for sepsis patients admitted in the ICU of 10 Chinese university hospitals was US$11,390 (2004–2005) [10]; and in France the mean cost of sepsis hospitalizations in the ICU was of €22,800 (1997–2000) [11].

Health indicators are essential to define strategies to improve the treatment of diseases, and, the epidemiology information of sepsis in developing countries is scarce [5,12–15]. Thus, the aim of this work is to assess trends in the incidence, lethality, and costs of sepsis for Brazilian Unified Health System (SUS–*Sistema Único de Saúde*) hospitalizations for the period from January 2006 to December 2015.

## Materials and methods

### Data sources

We used data from two databases available for public access from the DATASUS [16] website. DATASUS is the Informatics Department of the Brazilian Unified Health System. The first database is the Hospital Information System (SIHSUS–*Sistema de Informações Hospitalares do SUS*) that presents authorizations for hospital encounters (AIH–*Autorização de Internação Hospitalar*) performed under the SUS. Each AIH registry contains data from a hospital encounter: demographic information, hospital length of stay (LOS), costs, diagnoses, and patient hospital outcome [17]. No data present in this base have information that could identify patients. The second base is the National Registry of Healthcare Facilities (CNES–*Cadastro Nacional de Estabelecimentos de Saúde*), which is updated monthly and contains information about each facility. This database was used to define the size and type of hospitals (private or public), as well as the relation of intensive care unit (ICU) beds per total number of beds. The size of hospitals is defined according to the number of beds [18]: small hospitals have a maximum of 50 beds, medium hospitals have from 51 to 150 beds, large hospitals have from 151 to 500 beds, and very large hospitals have more than 500 beds.

### Selection of hospitalizations

As the SIHSUS database does not provide enough information to identify the presence of infection or organ dysfunction during a hospitalization, in this work we selected sepsis cases using a defined list of diagnosis codes as described in previous studies [4,13,19–21].

We selected AIH registries of patients with the primary diagnosis (most responsible diagnosis) of sepsis that had been hospitalized between 2006 and 2015. We used the list of sepsis diagnoses (ICD-10-CA, Canadian Revision) provided by the Canadian Institute for Health Information (CIHI) [20] (the complete list of diagnoses is available in S1 Appendix). Specific ICD-10-CA codes (A41.50, A41.51, A41.52, A41.58, A41.80, and A41.88) were not used, because they are not part of the SUS ICD-10 terminology. Registries of patients that had the primary diagnosis as one of the described in S1 Appendix were classified with sepsis and considered in our study.

### Data analysis

The SIHSUS data were processed to remove duplicated registries, to adjust the age of patients, and to deflate all costs to December 2015 using the Broad National Prices Index for the Consumer (IPCA—*Índice Nacional de Preços ao Consumidor Amplo)*[22]. All costs were converted to US dollars using the rate from December 2015 (R$1 was US$0.253) [23]. The costs presented in this work refer to what the government reimburses to hospitals for sepsis hospitalizations (including the payment of the hospital staff, hospital and intensive care unit accommodations, execution of procedures and exams). For intensive care unit (ICU) hospitalization costs, we considered only the hospitalization costs of registries that had the length of stay (LOS) in the ICU equal or greater than one day. For calculating the mean costs per case and the mean LOS we excluded the 5% extremes values to remove outliers. In this work, we considered hospital encounters that had discharge disposition as “discharge to home” or “death” since we wanted to extract the outcomes of the effectiveness of the treatment (patient lives or dies).

To calculate the sepsis incidence and mortality per 100,000 persons, we used the population projection by gender and age provided by the Brazilian Institute of Geography and Statistics (*Instituto Brasileiro de Geografia e Estatística*—IBGE)[24].

For race/ethnicity indicators, we considered data from 2008 to 2015 since there was no information available from 2006 and 2007. Regarding the hospital type, which can be private or public, for the public type, we considered federal, state, and municipal hospitals.

To understand the influence of age in the mortality rate, we created multiple logistic regression models using the patient age, gender and race as independent variables. For these analyses, we did not consider registries in which the race or gender was not informed.

The two-tailed Chi-Square test was applied for independent samples using nominal variables. To compare independent samples with continuous data (which were not normally distributed) we used the two-tailed Mann-Whitney U test. To calculate the correlation between LOS and costs, we applied the Pearson’s test. We considered the level of significance to be α = 0.05; that is, a result was statistically significant whenever p < 0.05. All statistical analyses were conducted using the R software [25].

## Results

**Fig 1:**
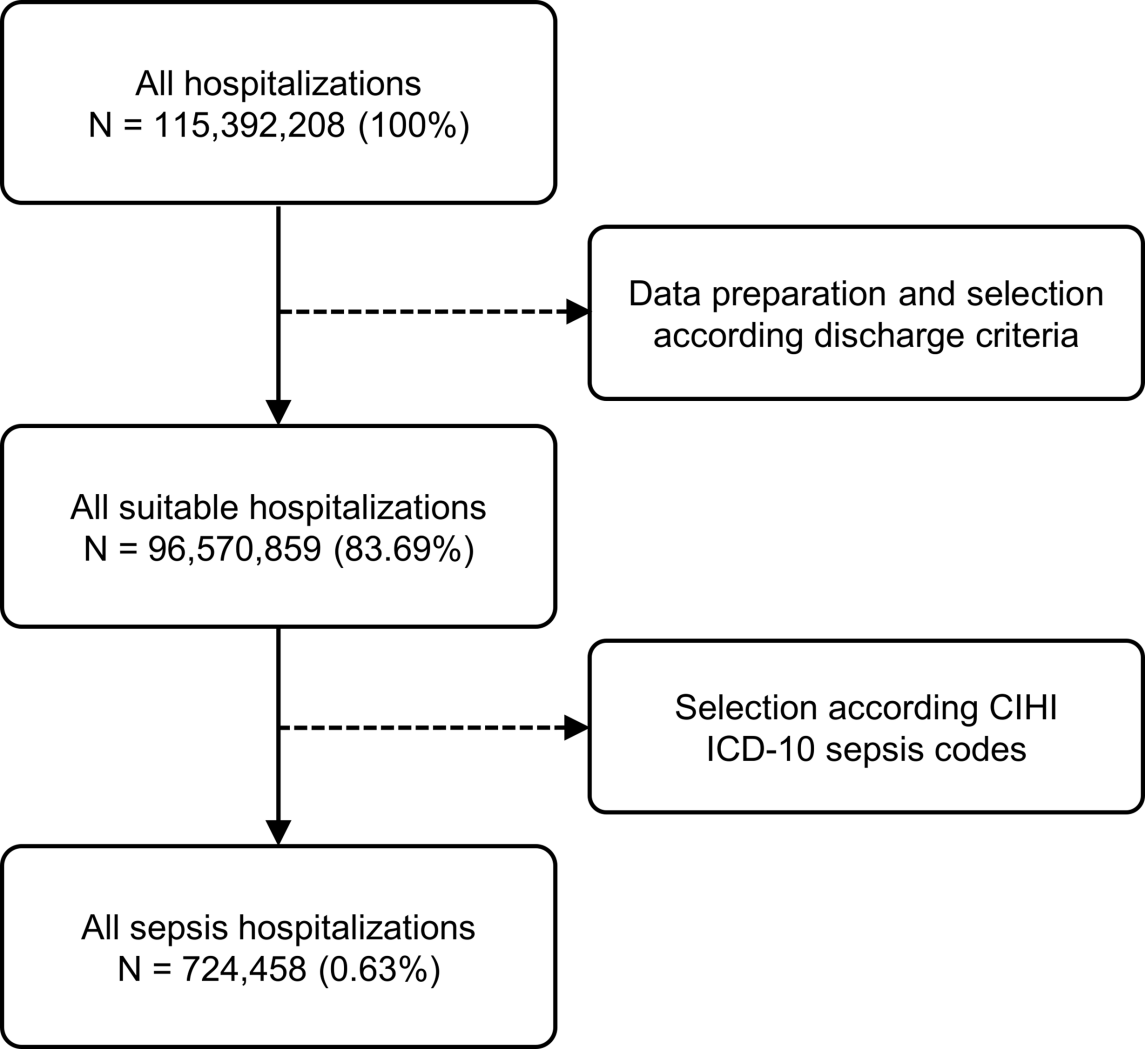
Flow diagram for a selection of sepsis cases. CIHI = Canadian Institute for Health Information; ICD-10 = International Statistical Classification of Diseases and Related Health Problems Tenth Revision.

From the original AIH database with 115,392,208 records, 96,570,859 (83.69%) were non-duplicated registries with discharge disposition as “discharge to home” or “death”, and 724,458 (0.63%) records were of hospitalizations with the primary diagnosis of sepsis. The flow diagram for the selection of sepsis cases can be found in Fig 1. These sepsis cases were treated in 4,271 different Brazilian hospitals.

**Fig 2:**
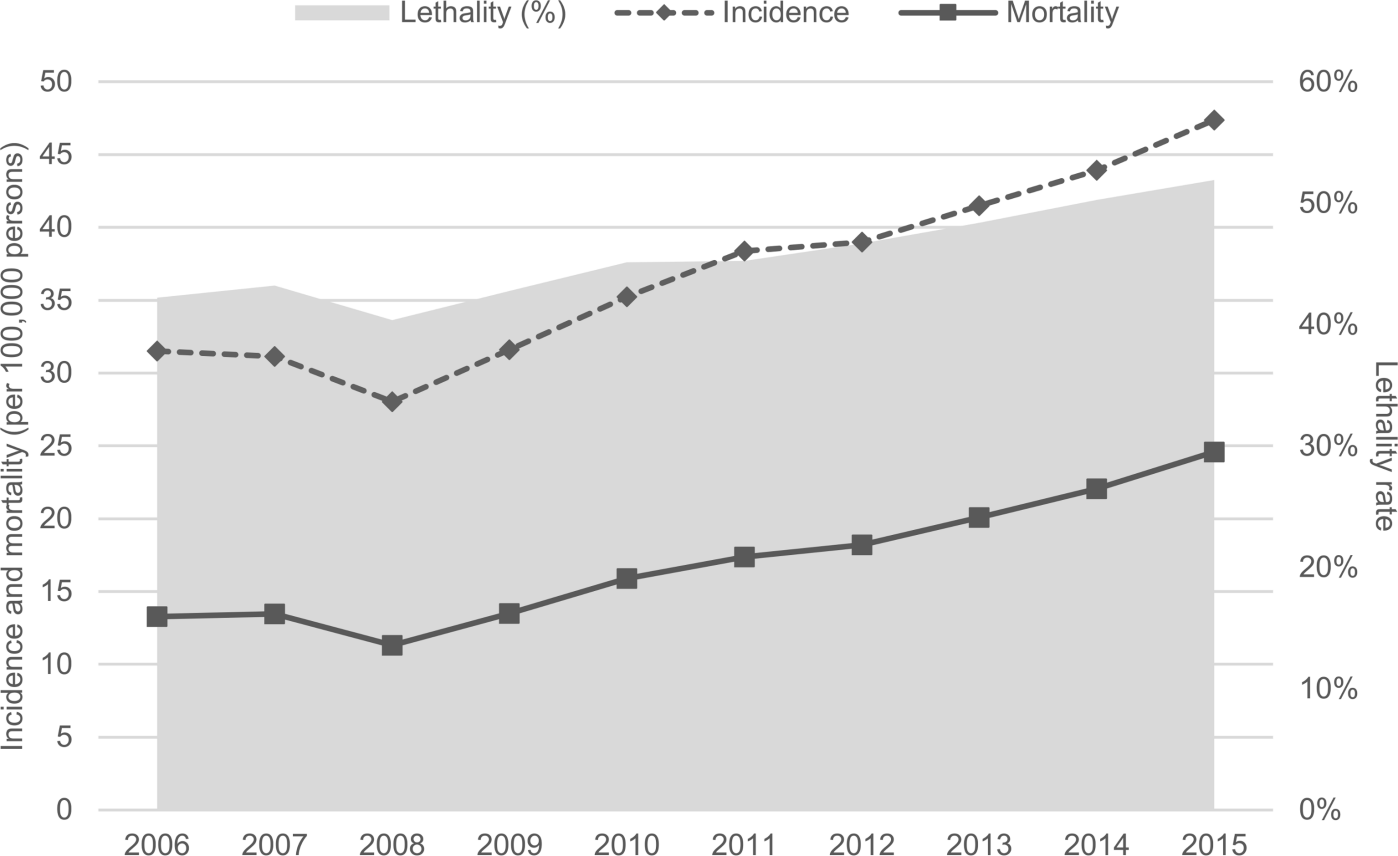
Incidence, mortality (per 100,000 persons) and lethality of sepsis from 2006 to 2015.

Fig 2 shows the incidence, lethality and mortality from 2006 to 2015. During this period, the incidence of sepsis increased 50.5% from 31.5/100,000 to 47.4/100,000 persons per year. The number of sepsis cases over the total number of cases (considering all diseases that had discharge disposition as “discharge to home” or “death”) was 0.75% for the period.

Table 1 presents the number of sepsis cases by patient characteristics from 2006 to 2015 grouped every 2 years. During this period, the average proportion of cases for female patients was 47.7%. Regarding age, the group of adults (from 18 to 64 years old) had the highest proportion of sepsis, which represented 32.5% of cases, followed by the children/teenagers group (from 0 to 17 years old) with 29.8% of cases. The number of sepsis cases for children/teenagers decreased 5.7%, and for the adults, the elderly (from 65 to 84 years old), and the very old (greater than 85 years old), it increased 68.8%, 135.0%, and 205.8%, respectively. The mean patient age was 45.2 years old increasing 34.8% (from 38.2 to 51.5 years old) during the study time frame. Regarding race, white and brown (mixed-race) subjects were the majority of individuals representing 36.8% and 26.2% of cases, respectively; black, yellow (Asian), and indigenous races represented 3.9% of cases. 33.1% of cases had no information with respect to race.

**Table 1:** Number of sepsis cases by patient characteristics from 2006 to 2015.

Indicator / Attribute	2006–2007 N	2008–2009 N	2010–2011 N	2012–2013 N	2014–2015 N	Average N (%)	Annual Growth Percentage^b^
Total							
Cases of sepsis	118,014	114,834	144,636	161,100	185,874	72,446^c^	64.1%
% from all hospitalizations	0.60%	0.59%	0.74%	0.85%	0.99%	0.75%	76.3%
Cases per gender							
Male	62,098	60,413	75,742	84,263	96,553	37,907^c^ (52.3%)	60.8%
Female	55,915	54,421	68,894	76,837	89,321	34,539^c^ (47.7%)	67.9%
Cases per age							
0–17	43,920	40,012	44,860	44,699	42,708	21,620^c^ (29.8%)	-5.7%
18–64	37,964	37,763	46,998	52,523	60,503	23,575^c^ (32.5%)	68.8%
65–84	28,865	29,440	41,215	49,056	62,247	21,082^c^ (29.1%)	135.0%
85+	7,265	7,619	11,563	14,822	20,416	6,169^c^ (8.5%)	205.8%
Mean patient age in years	39.1	40.9	44.4	47.0	50.8	45.2	34.8%
Race/Ethnicity^a^							
White	NA	43,810	54,067	58,044	67,345	27,908^c^ (36.8%)	69.2%
Black	NA	3,605	4,733	4,961	5,936	2,404^c^ (3.2%)	97.4%
Brown (Mixed-race)	NA	25,475	33,953	42,899	56,578	19,863^c^ (26.2%)	163.1%
Yellow (Asian)	NA	481	591	653	1,296	378^c^ (0.5%)	355.6%
Indigenous	NA	439	301	246	278	158^c^ (0.2%)	-45.4%
No Information	NA	41,024	50,991	54,297	54,441	25,094^c^ (33.1%)	41.0%
Death cases	50,388	47,799	65,359	76,638	95,009	33,519^c^	101.9%

^a^Following the nomenclature and order presented in SIHSUS database.

^b^Growth percentage from 2006 to 2015.

^c^Annual average.

NA = not available.

Table 2 presents the incidence of sepsis per 100,000 persons. From 2006 to 2015, the incidence for female patients increased 53.2% from 29.5/100,000 to 45.2/100,000 persons per year and for male patients, it increased 47.6% from 33.6/100,000 to 49.6/100,000. Regarding age, the group of very old people had the highest incidence of sepsis, which represented 517.6/100,000 persons, followed by the elderly group at 169.0/100,000 persons. The sepsis incidence for children/teenagers increased 0.5%, and for the adults, the elderly, and the very old, it increased 47.2%, 72.2%, and 86.7%, respectively.

**Table 2:** Incidence and mortality per 100,000 persons, and lethality rates by patient characteristics from 2006 to 2015.

Indicator / Attribute	2006–2007 N	2008–2009 N	2010–2011 N	2012–2013 N	2014–2015 N	Average	Annual Growth Percentage^a^
Incidence	31.3	29.8	36.8	40.2	45.6	36.9	50.5%
Incidence per gender							
Male	33.3	31.7	39.0	42.6	48.0	39.1	47.6%
Female	29.4	28.0	34.7	38.0	43.3	34.8	53.2%
Incidence per age groups							
0–17	35.7	32.9	37.4	37.8	36.8	36.1	0.5%
18–64	16.5	15.8	19.1	20.8	23.3	19.2	47.2%
65–84	132.6	127.5	167.1	185.0	217.0	169.0	72.2%
85+	384.1	363.7	493.5	561.8	692.3	517.6	86.7%
Mortality	13.4	12.4	16.6	19.1	23.3	17.1	85.0%
Lethality	42.7%	41.6%	45.2%	47.6%	51.1%	46.3%	23.0%
Lethality for ICU hospitalizations	60.7%	61.7%	63.0%	65.2%	68.4%	64.5%	14.4%

^a^Growth percentage from 2006 to 2015.

Table 3 shows the number of sepsis cases per type and size of hospital. The average proportion of cases for private hospitals was 49.9%. During the study period, the number of sepsis hospitalizations for private hospitals increased 33.0% and for public hospitals, increased 103.5%. With respect to the size of hospitals, large hospitals treated the highest proportion of sepsis cases (47.8%) followed by medium (33.0%), very large (10.1%) and small hospitals (9.1%). From 2006 to 2015, the number of sepsis cases for small, medium, large and very large hospitals increased 15.7%, 41.8%, 94.5% and 74.4%, respectively.

**Table 3:** Number of sepsis cases and lethality rates per hospital type and size from 2006 to 2015.

Indicator / Attribute	2006–2007 N	2008–2009 N	2010–2011 N	2012–2013 N	2014–2015 N	Average N (%)	Annual Growth Percentage^a^
Cases per type of hospital							
Private	64,787	61,373	72,187	78,655	84,196	36,120^b^ (49.9%)	33.0%
Public	53,062	53,416	72,440	82,441	101,675	36,303^b^ (50.1%)	103.5%
Death cases per type of hospital							
Private	23,132	19,280	24,475	29,806	36,868	13,356^b^ (39.9%)	68.8%
Public	27,188	28,511	40,880	46,831	58,141	20,155^b^ (60.1%)	131.3%
Lethality per type of hospital							
Private	35.7%	31.4%	33.9%	37.9%	43.8%	37.0%	26.9%
Public	51.2%	53.4%	56.4%	56.8%	57.2%	55.5%	13.6%
Cases per size of hospital							
Small	12,892	10,989	12,892	14,096	14,899	6,577^b^ (9.1%)	15.7%
Medium	41,704	39,309	48,091	51,892	57,964	23,896^b^ (33.0%)	41.8%
Large	51,628	52,870	70,011	78,935	92,945	34,639^b^ (47.8%)	94.5%
Very large	11,430	11,571	13,633	16,173	20,063	7,287^b^ (10.1%)	74.4%
Death cases per size of hospital							
Small	2,625	2,340	2,848	3,407	3,859	1,508^b^ (4.5%)	59.5%
Medium	15,109	13,258	19,152	21,545	25,888	9,495^b^ (28.3%)	83.1%
Large	25,691	24,334	34,325	41,777	52,800	17,893^b^ (53.4%)	125.6%
Very large	6,822	7,855	9,030	9,908	12,462	4,608^b^ (13.8%)	79.5%
Lethality per size of hospital							
Small	20.4%	21.3%	22.1%	24.2%	25.9%	22.9%	38.2%
Medium	36.2%	33.7%	39.8%	41.5%	44.7%	39.7%	29.3%
Large	49.8%	46.0%	49.0%	52.9%	56.8%	51.7%	16.1%
Very large	59.7%	67.9%	66.2%	61.3%	62.1%	63.2%	3.0%

^a^Growth percentage from 2006 to 2015.

^b^Annual average.

From 2006 to 2015, the mortality due to sepsis increased 85.0%, going from 13.3/100,000 to 24.6/100,000 persons per year. The overall lethality rate of sepsis was 46.3%, and for hospitalizations with admission to the ICU, it was 64.5%. The number of sepsis deaths over the total number of deaths (all admissions) was 8.2% for the period. The average lethality rate for female patients (46.8%) was higher than male patients (45.8%) (Chi-square test results: χ^2^ = 77.9; df = 1; p < 0.001; two-tailed). With respect to the age of patients, very old patients had the highest lethality rate of 75.9% followed by the elderly, the adults, and the children/teenagers groups with 67.7%, 49.3%, and 13.6%, respectively (χ^2^ = 154,830; df = 3; p < 0.001; two-tailed). The lethality rate for children/teenagers decreased 40.1% and for adults, elderly and very old, it increased 11.5%, 6.1%, and 2.8%, respectively. Regarding the race/ethnicity, indigenous and brown (mixed-race) patients had the smallest lethality rate of 30.1% and 42.1%, respectively, while black, yellow (Asian), and white patients had the rate of 52.0%, 51.6%, and 49.9%, respectively (χ^2^ = 2,643.9; df = 4; p < 0.001; two-tailed). The sepsis lethality rate in public hospitals (55.5%) was higher than private hospitals (37.0%) (χ^2^ = 25,036; df = 1; p < 0.001; two-tailed). Regarding the size of hospitals, small hospitals had the smallest lethality rate with an average of 22.9%, medium hospitals had an average of 39.7%, large hospitals had 51.7%, and very large hospitals had 63.2% (χ^2^ = 30,991; df = 3; p < 0.001; two-tailed).

**Fig 3:**
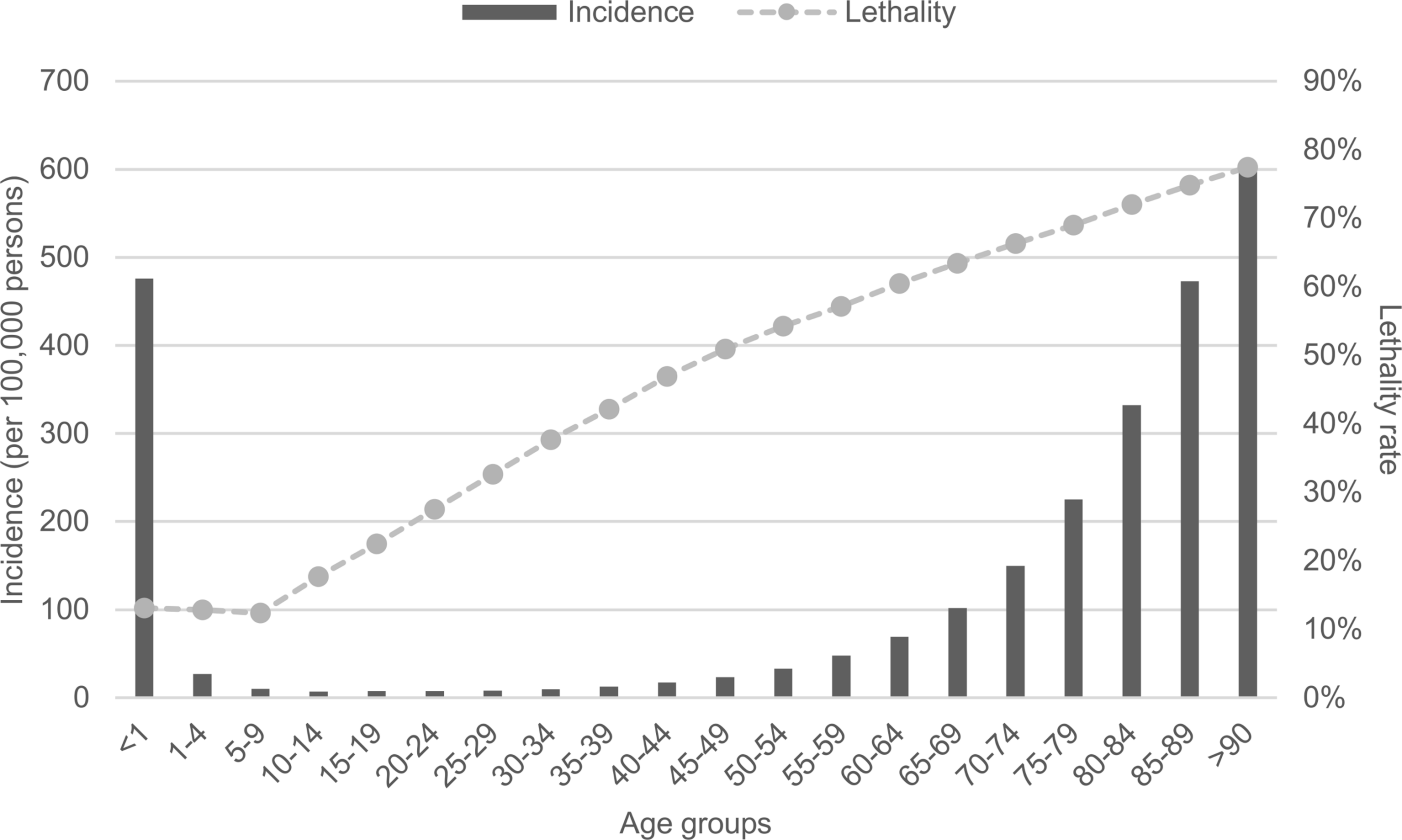
Sepsis incidence (per 100,000 persons) and lethality rate according to the age groups.

Fig 3 presents the sepsis incidence (per 100,000 persons) and lethality according to each age group. Analyzing the figure, we can note that children younger than 1-year-old had a high incidence of sepsis (475.9 cases per 100,000 persons) with a lower lethality rate (13.1%). Children in the group of 5–9 years old had the lowest lethality rate (12.3%). In contrast, people more than 90 years old had the highest incidence of sepsis (601.4 cases per 100,000 persons) with the highest lethality rate (77.5%). Performing multiple logistic regressions (death as a dependent variable, and age, gender, and race as independent variables), we observed that age has a strong association with mortality. When the age variable is removed, the prediction error increases from 29.7% to 46.7%. Performing a logistic regression with death as a dependent variable and age as an independent variable, the prediction error was 29.7%. All ROC curves are available in S2 Appendix. Analyzing the results of the last logistic regression, we conclude that the older the patient is, the higher the probability is to die when diagnosed with sepsis (for 1-year change in age, the odds to die increases 1.036 times).

Regarding race lethality, brown (mixed-race) and indigenous races had the lowest lethality rates (42.1% and 30.1%, respectively). Comparing the age of brown and indigenous races with white, black, and yellow (Asian) races, the age is significantly lower for brown (mean 40.1 years; median 46 years) (Mann-Whitney U test results: U = 15,572,966,908; n1 = 159,680; n2 = 247,170; p < 0.001; two-tailed) and for indigenous patients (mean 17.0 years; median 1 year) (U = 67,934,676; n1 = 1,281; n2 = 247,170; p < 0.001; two-tailed) than for the other races (mean 52.4 years; median 61 years). Female patients had in average higher lethality rates (46.8%) than male patients (45.8%). Comparing the age of each gender, male patients were in average younger (mean 43.7 years; median 52 years) than female patients (mean 46.9 years; median 56 years) (U = 60,897,122,525.5; n1 = 379,069; n2 = 345,388; p < 0.001; two-tailed). Thus, as brown and indigenous patients, in general, were younger than the other race groups, as well as, male patients were younger than female patients, we can consider that age is an important attribute to explain these differences in lethality rates.

Table 4 presents hospitalizations, costs, and LOS grouped every 2 years. The mean cost per hospitalization was US$624.0 (median US$353.9) with a growth percentage of 23.5% between 2006 and 2015, and the mean cost per ICU hospitalization was US$1,708.1 (median US$1,293.1) with a growth percentage of 42.2%. The mean hospitalization LOS was 9.0 days (median 7.0 days), while the mean LOS in the ICU was 8.0 days (median 6.0 days). From 2006 to 2015, the LOS decreased 2.2% going from 9.3 to 9.1 days and the ICU LOS increased 5.2% going from 7.7 to 8.1 days.

**Table 4:** Hospitalizations, costs and LOS of sepsis from 2006 to 2015.

Indicator / Attribute	2006–2007 N (SD)	2008–2009 N (SD)	2010–2011 N (SD)	2012–2013 N (SD)	2014–2015 N (SD)	Average	Annual Growth Percentage^c^
All hospitalizations	118,014	114,834	144,636	161,100	185,874	72,446^d^	64.1%
ICU hospitalizations	33,466	30,543	40,477	47,894	58,542	21,092^d^	83.9%
% ICU hospitalizations	28.4%	26.6%	28.0%	29.7%	31.5%	29.1%	12.0%
Hospitalizations costs^a^							
Total cost (US$ million)	78.2	104.4	133.1	143.5	156.1	61.5^d^	113.3%
Mean cost per case (US$)^b^	512.6 (420.6)	658.7 (683.7)	669.6 (701.8)	648.1 (687.2)	619.2 (662.2)	624.0	23.5%
Costs for hospitalizations requiring ICU^a^							
Total cost (US$ million)	48.2	71.0	93.0	103.0	114.1	42.9^d^	159.0%
Mean cost per case (US$)^b^	1,220.2 (838.3)	1,928.9 (1,457.5)	1,927.5 (1,416.9)	1,808.8 (1,325.6)	1,657.1 (1,195.6)	1,708.1	42.2%
Mean LOS							
Hospitalization (days)^b^	9.3 (7.5)	8.8 (7.4)	8.6 (7.3)	9.0 (7.7)	9.1 (7.6)	9.0	-2.2%
ICU (days)^b^	7.8 (6.7)	8.1 (7.0)	7.8 (6.7)	8.0 (6.8)	8.2 (6.8)	8.0	5.2%

^**a**^Deflated costs (December 2015, IPCA)

^**b**^5% extremes excluded

^**c**^Growth percentage from 2006 to 2015

^d^Annual average.

SD = standard deviation

The mean hospitalization LOS for public hospitals was higher than private hospitals, with a difference of 35.5% ranging from 7.6 days (median 6.0 days) to 10.3 days (median 8.0 days) (U = 51,666,193,009; n1 = 344,310; n2 = 345,562; p < 0.001; two-tailed). Public hospitals had a higher mean ICU LOS than private hospitals, with a difference of 26.8% ranging from 7.1 days (median 5.0 days) to 9 days (median 7.0 days) (U = 4,388,877,014.5; n1 = 102,992; n2 = 97,714; p < 0.001; two-tailed).

The average daily hospitalization cost was US$94.4 (median US$66.1) for private hospitals, and US$90.3 (median US$58.4) for public ones (U = 54,091,530,825; n1 = 344,893; n2 = 342,978; p < 0.001; two-tailed). The mean case cost was US$586.2 (median US$343.6) for private hospitals, and US$662.9 (median US$372.4) for public hospitals (U = 57,418,114,611.5; n1 = 347,283; n2 = 345,789; p < 0.001; two-tailed). Related to the average cost per case, public hospitals had higher costs than private hospitals mainly because the LOS and the ICU LOS in public hospitals were higher. The Pearson’s correlation coefficient between LOS and costs was 0.58 (Pearson’s correlation test results: t = 608.26; df = 724,460; p < 0.001; two-tailed), indicating a moderate positive relationship, and the Pearson’s correlation coefficient between ICU LOS and costs was 0.92 (t = 2,000.4; df = 724,460; p < 0.001; two-tailed), indicating a strong positive relationship.

Analyzing lethality rates, LOS, and mean cost per case, we can observe that, in general, private hospitals have a more effective treatment for sepsis compared with the public hospitals. This means that private hospitals treat patients faster than public hospitals, with lower total costs and lower lethality rates. We observed a small difference in the average age of patients that were treated in private (44.8 years; median 53 years) and public hospitals (45.7 years; median 55 years) (U = 64,847,829,992.5; n1 = 361,198; n2 = 363,034; p < 0.001; two-tailed). Performing a logistic regression for each type of hospital with death as the dependent variable and age as the independent variable, we observed that the age has similar partial slope coefficients for both cases (private with 0.0337 and public with 0.0382). Thus, the age variable does not explain the inefficiency in public hospitals. Nevertheless, we cannot exclude possible differences in the type of patients admitted and the severity of illness between public and private hospitals, as we did not have access to this sort of information. During the study period, the percentage of ICU beds (total ICU beds/total beds) in private hospitals (6.0%) was close to the percentage in public hospitals (5.5%) (U = 2,498,347,306.5; n1 = 83,138; n2 = 60,843; p < 0.001; two-tailed), evidencing that both types of hospitals had similar resources to monitor and treat severe patients.

**Fig 4:**
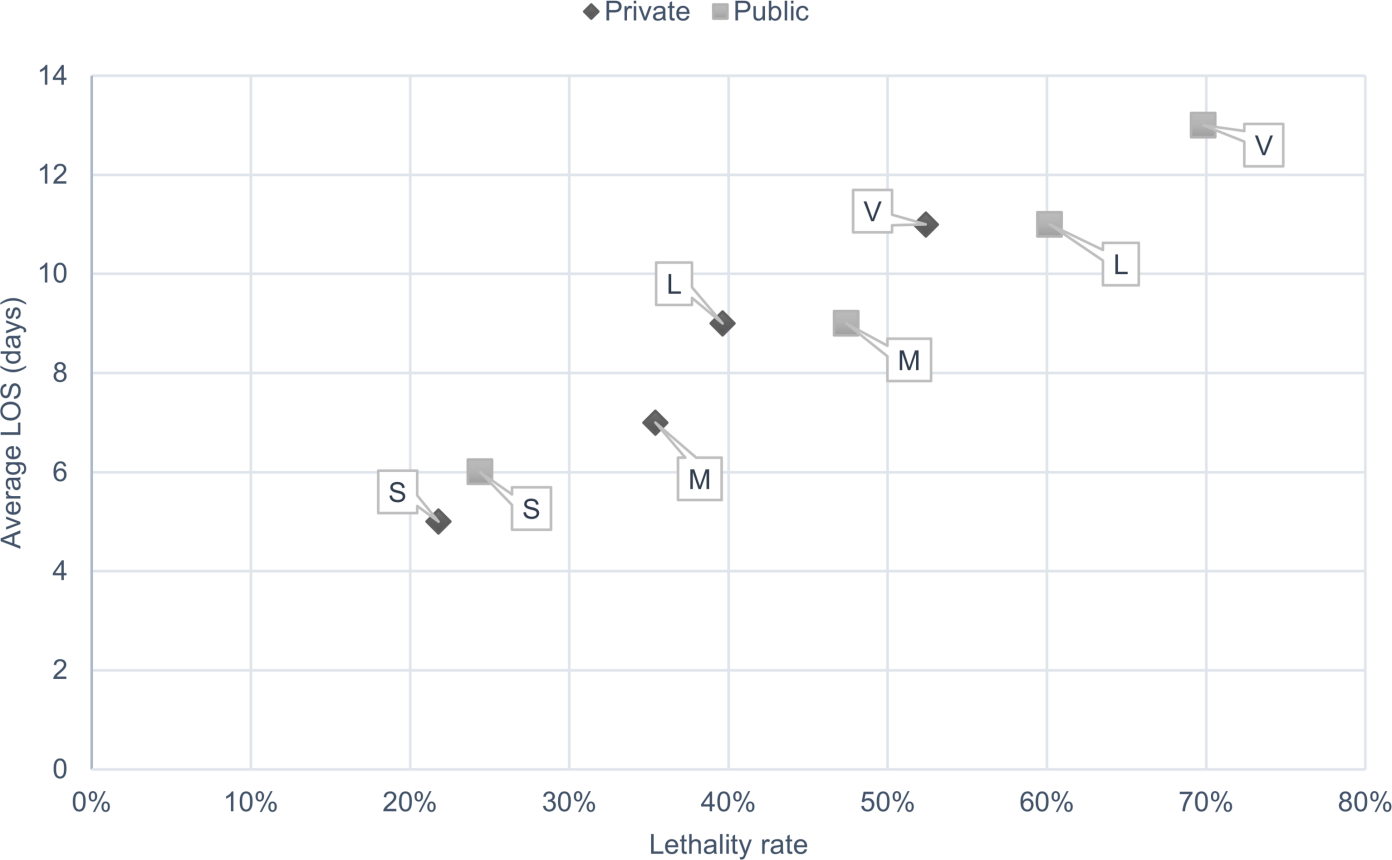
Treatment efficiency matrix for sepsis per hospital size and type. Letters refer to the size of hospitals: M = medium size; L = large size; S = small size; V = very large size. LOS = length of stay.

To give a general overview of the efficiency of hospitals for treating sepsis, Fig 4 presents an efficiency matrix. We created it inspired in the efficiency matrix presented by Salluh et al. [26]. Each dot is a group of hospitals of the same size and type. Each dot was added in the matrix according to its average LOS (Y-axis) and average lethality rate (X-axis). The letter near each dot represents the size of the hospital group (S = small, M = medium, L = large, V = very large). The shape of the dot represents the type of hospital (diamond shapes are private, square shapes are public). Hospital groups that are located in the bottom left part of the matrix are more efficient than the groups that are located in the top right. We can observe that smaller hospitals are more efficient than larger hospitals and private hospitals are more efficient than public ones. The number of hospitals and cases per hospital group can be found in S3 Appendix.

## Discussion

We observed that the number and incidence of SUS sepsis hospitalizations increased from 2006 to 2015. This phenomenon can be related to population aging. According to the World Health Organization [27], Brazilian life expectancy has grown from 73 years to 75 years (from 2006 to 2015), which has increased the older people groups [28]. During the study period, the mean age of septic patients in the current research study increased from 38.2 to 51.5 years old. Another reason can be associated to initiatives like the Surviving Sepsis Campaign [29,30], which could improve the awareness of health professionals to provide the right diagnosis of sepsis [2].

Analyzing the multiple logistic regressions results, age presented a high association with patients’ death (for 1-year change in age, the odds to die increases 1.036 times). From 2006 to 2015, the incidence of sepsis cases for the elderly and the very old age groups increased 72.2% and 86.7%, respectively, and these two groups had the highest average lethality rate of 67.7% and 75.9%, respectively. Nevertheless, during the study period, we observed that the lethality rate for adults, elderly and very old increased 11.5%, 6.1%, and 2.8%, respectively, indicating that age is not the only factor for worsening mortality.

The fact that the lethality rate in public hospitals (55.5%) was higher than private ones (37.0%), can be a result of delayed sepsis recognition and treatment, or fewer resources in the public hospitals than in the private ones, as it has been previously published [31]. The different characteristics in care provided by each type of hospital, the structure of the ICU health team, the delay in transferring the patient to the ICU, and the access to the best standard care may contribute to these different rates in mortality [12].

The overall sepsis lethality rate was 46.3%, and for hospitalizations with admission to the ICU, it was 64.5%. The mortality rates presented in studies for countries with a similar Human Development Index (HDI) (2014) [32] to Brazil (HDI 0.755) were: China (HDI 0.727) with 33.5–48.7% for sepsis (multicenter investigations in ICUs) [33]; Colombia (HDI 0.720) with 21.9% for sepsis, and 45.6% for septic shock (multicenter investigation of cases of patients admitted in the emergency department, general wards and ICUs) [34]; and Mexico (HDI 0.756) with 30.4% (multicenter investigation in ICUs) [35]. The lethality rates presented in the current study are higher than the lethality rates presented above, even when comparing to countries with a lower HDI, such as Colombia and China.

Concerning to hospitals costs, the mean cost per hospitalization was US$624.0 (median US$353.9) and per ICU hospitalization was US$1,708.1 (median US$1,293.1). Applying the ratio of the purchasing power parity (PPP) conversion factor to the market exchange rate [36], the cost was US$1,040.0 (median US$589.8) per hospitalization and US$2,846.8 (median US$2,155.2) per ICU hospitalization. As Sogayar et al. [9] discussed, a simple comparison between studies is not easy, since reimbursement rates, costs, price factors, and healthcare systems may vary. Nevertheless, the values presented in the current work are low if we compare them with the costs of other sepsis studies. Chalupka and Talmor [8] presented that the costs per sepsis case had a wide variability ranging from US$4,888 (Argentina) to US$103,529 (United States). Comparing the median ICU hospitalization costs (US$2,155.2 –adjusted costs using the ratio of PPP) of the current research study (2006–2015), that represent the government reimbursement to hospitals for treating sepsis, with the median case costs of 21 ICUs of private and public Brazilian hospitals (US$9,632) presented by Sogayar et al. [9] (2003–2004), one may suppose that the reimbursement values are lower than the effective costs. According to Victora et al. [37], in Brazil, private institutions debate that the reimbursement provided by SUS barely allows them to cover all costs. The SUS’s low reimbursement to hospitals for treating sepsis may be one of the reasons for the high lethality rates.

Even though this work could provide 10 years of sepsis indicators, it has limitations. As we had no access to clinical variables (type of infection, severity scores, vital signs, laboratory exam results, co-morbidities), we could not improve the regression models to have a more precise estimative, we could not verify the accuracy in the selection of cases, and we could not define the severity of the sepsis (definition of case-mix). Jolley et al. [38] performed a research study using data from three Canadian hospitals to identify the sensitivity and specificity in selecting septic patients using the CIHI ICD-10 codes. The sensitivity was 46.4% and the specificity was 98.7% for selecting ICU adult septic patients. For selecting non-ICU septic patients, the sensitivity was 6.7% and the specificity was 100%. These results indicate that in our study we underestimated the total number of sepsis cases.

It was a challenge for us to define the right list of ICD-10 codes to use in this study since there is no official list. There are several lists of ICD-10 codes used in different studies [39]. In addition, as Tsertsvadze et al. [40] presented, it is difficult to determine the true sepsis incidence of a population because there is an absence of valid standard methods for defining sepsis. Thus, we consider it is important to create a unique list of sepsis diagnosis codes and a standard approach for selecting sepsis cases. This would provide the generation of homogenized indicators, which would allow appropriate comparisons and would encourage health professionals to use the right diagnosis codes.

More than 75% of the Brazilian population depends on and exclusively uses the SUS health services [41]. The rest of the population has access to private health services, but they can also use the SUS services since they are available to any person. Thus, since we could not obtain the exact percentage of users that exclusively accessed private health services, we decided to use the Brazilian population projection [24] without any adjustment to generate the sepsis incidence and mortality per 100,000 persons.

## Conclusions

The incidence of SUS sepsis hospitalizations increased 50.5% in Brazil during the period from 2006 to 2015. The overall lethality rate of sepsis was 46.3%, and for hospitalizations with admission to the ICU, it was 64.5%. During the study period, the lethality rate for children/teenagers improved, but for all other age groups it became worse with an increase of 11.4%. The lethality rate in public hospitals (55.5%) was higher than in private hospitals (37.0%), which possibly reflects the differences in the number of resources for processes and/or structure. The SUS’s low reimbursement to hospitals for treating sepsis may be one of the reasons for the high lethality rates.

This work, differently from previous studies [9,13,31,42], characterizes the epidemiology of sepsis over 10 years using a national database, including all hospitalizations (not only intensive care unit cases), all severities of sepsis, and all patient ages (not only adults), thus, providing a broad overview of sepsis in Brazil. We expect that this work, presenting the reality of a middle-income country, may help international organizations (e.g. the Society of Critical Care Medicine) in planning policies to improve the sepsis worldwide scenario.

## Supporting information

S1 Appendix The Canadian Institute for Health Information list of International Statistical Classification of Diseases and Related Health Problems Tenth Revision (ICD-10) codes used to define sepsis. (DOCX)

S2 Appendix ROC curves from multiple logistic regressions. (DOCX)

S3 Appendix Number of hospitals and cases per hospital group from the treatment efficiency matrix for sepsis. (DOCX)

S4 Appendix Process to get SIHSUS and CNES data from DATASUS. (DOCX)
