# Supplementary material for: Epidemiology of sepsis in Brazil: Incidence, lethality, costs, and other indicators for Brazilian Unified Health System hospitalizations from 2006 to 2015
Source: PLoS One. 2018 Apr 13;13(4):e0195873. doi: 10.1371/journal.pone.0195873 (PMC5898754; doi:10.1371/journal.pone.0195873)
Supplement: S1 Appendix — (DOCX) [file pone.0195873.s001.docx]

**S1 Appendix. The Canadian Institute for Health Information list of International Statistical Classification of Diseases and Related Health Problems Tenth Revision (ICD-10) codes used to define sepsis**

| **ICD-10 code** | **Description** |
| --- | --- |
| A02.1 | Salmonella sepsis |
| A03.9 | Shigellosis, unspecified |
| A20.7 | Septicaemic plague |
| A21.7 | Generalized tularaemia |
| A22.7 | Anthrax sepsis |
| A23.9 | Brucellosis, unspecified |
| A24.1 | Acute and fulminating melioidosis |
| A26.7 | Erysipelothrix sepsis |
| A28.0 | Pasteurellosis |
| A28.2 | Extraintestinal yersiniosis |
| A32.7 | Listerial sepsis |
| A39.2 | Acute meningococcaemia |
| A39.3 | Chronic meningococcaemia |
| A39.4 | Meningococcaemia, unspecified |
| A40 | Streptococcal sepsis |
| A40.0 | Sepsis due to streptococcus, group A |
| A40.1 | Sepsis due to streptococcus, group B |
| A40.2 | Sepsis due to streptococcus, group D |
| A40.3 | Sepsis due to Streptococcus pneumoniae |
| A40.8 | Other streptococcal sepsis |
| A40.9 | Streptococcal sepsis, unspecified |
| A41 | Other sepsis |
| A41.0 | Sepsis due to Staphylococcus aureus |
| A41.1 | Sepsis due to other specified staphylococcus |
| A41.2 | Sepsis due to unspecified staphylococcus |
| A41.3 | Sepsis due to Haemophilus influenzae |
| A41.4 | Sepsis due to anaerobes |
| A41.5 | Sepsis due to other Gram-negative organisms |
| A41.8 | Other specified sepsis |
| A41.9 | Sepsis, unspecified |
| A42.7 | Actinomycotic sepsis |
| B00.7 | Disseminated herpesviral disease |
| B37.7 | Candidal sepsis |
| P35.2 | Congenital herpesviral [herpes simplex] infection |
| P36 | Bacterial sepsis of newborn |
| P36.0 | Sepsis of newborn due to streptococcus, group B |
| P36.1 | Sepsis of newborn due to other and unspecified streptococci |
| P36.2 | Sepsis of newborn due to Staphylococcus aureus |
| P36.3 | Sepsis of newborn due to other and unspecified staphylococci |
| P36.4 | Sepsis of newborn due to Escherichia coli |
| P36.5 | Sepsis of newborn due to anaerobes |
| P36.8 | Other bacterial sepsis of newborn |
| P36.9 | Bacterial sepsis of newborn, unspecified |
| P37.2 | Neonatal (disseminated) listeriosis |
| P37.5 | Neonatal candidiasis |
