# Supplementary material for: Epidemiology of sepsis in Brazil: Incidence, lethality, costs, and other indicators for Brazilian Unified Health System hospitalizations from 2006 to 2015
Source: PLoS One. 2018 Apr 13;13(4):e0195873. doi: 10.1371/journal.pone.0195873 (PMC5898754; doi:10.1371/journal.pone.0195873)
Supplement: S2 Appendix — (DOCX) [file pone.0195873.s002.docx]

**S2 Appendix. ROC curves from multiple logistic regressions**


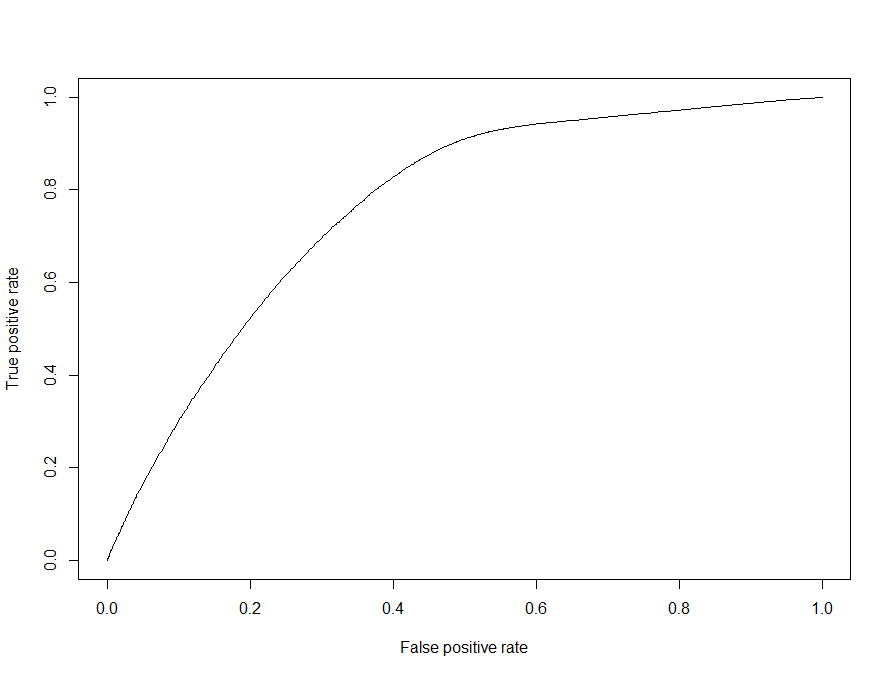


**Fig A. ROC Curve - death as dependent variable and age, gender, race as independent variables**. The prediction error is 29.7% and the area under the ROC curve is 0.76


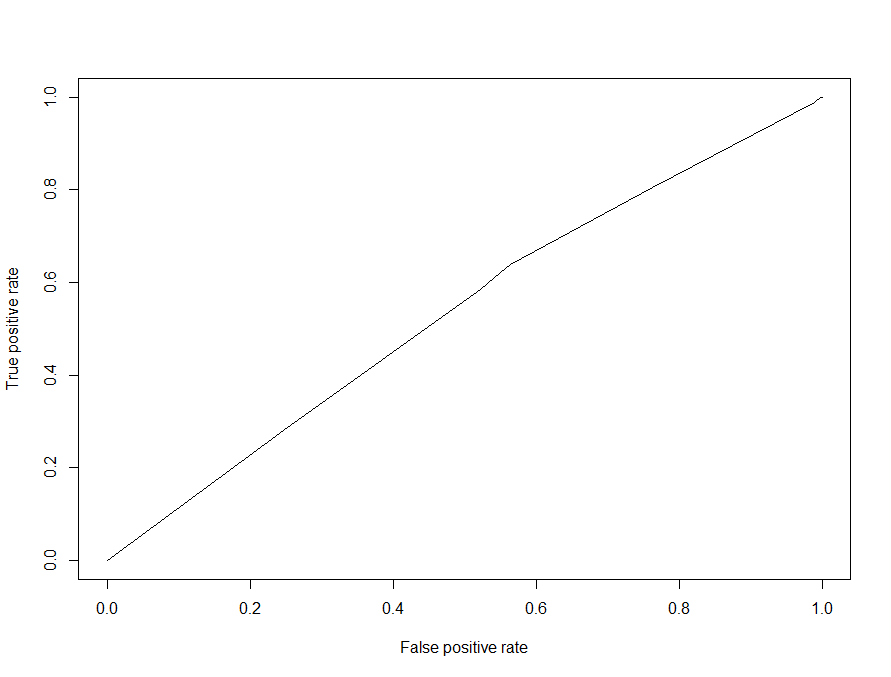


**Fig B. ROC Curve - death as dependent variable and gender, race as independent variables.** The prediction error is 46.7% and the area under the ROC curve is 0.54


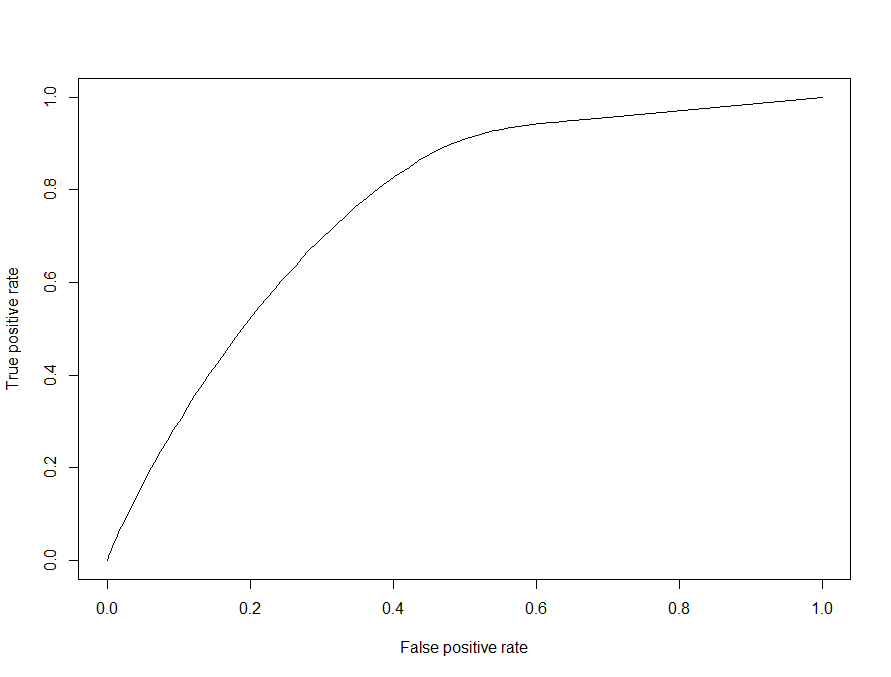


**Fig C. ROC Curve - death as dependent variable and age as independent variable.** The prediction error is 29.7% and the area under the ROC curve is 0.76
