# Supplementary material for: Epidemiology of sepsis in Brazil: Incidence, lethality, costs, and other indicators for Brazilian Unified Health System hospitalizations from 2006 to 2015
Source: PLoS One. 2018 Apr 13;13(4):e0195873. doi: 10.1371/journal.pone.0195873 (PMC5898754; doi:10.1371/journal.pone.0195873)
Supplement: S3 Appendix — (DOCX) [file pone.0195873.s003.docx]

**S3 Appendix. Number of hospitals and cases per hospital group from the treatment efficiency matrix for sepsis**

**Number of cases per hospital group**

| Hospital Type | Hospital Size | Number of Cases |
| --- | --- | --- |
| private | small | 37,111 |
| private | medium | 152,771 |
| private | large | 143,608 |
| private | very large | 27,548 |
| public | small | 28,657 |
| public | medium | 86,189 |
| public | large | 202,781 |
| public | very large | 45,322 |

**Number of hospitals per hospital group**

| Hospital Type | Hospital Size | Number of Hospitals |
| --- | --- | --- |
| private | small | 1,111 |
| private | medium | 1,282 |
| private | large | 368 |
| private | very large | 26 |
| public | small | 1,304 |
| public | medium | 707 |
| public | large | 322 |
| public | very large | 32 |
