# Supplementary material for: Epidemiology of sepsis in Brazil: Incidence, lethality, costs, and other indicators for Brazilian Unified Health System hospitalizations from 2006 to 2015
Source: PLoS One. 2018 Apr 13;13(4):e0195873. doi: 10.1371/journal.pone.0195873 (PMC5898754; doi:10.1371/journal.pone.0195873)
Supplement: S4 Appendix — (DOCX) [file pone.0195873.s004.docx]

**S4 Appendix. Process to get SIHSUS and CNES data from DATASUS**

To get SIHSUS data from DATASUS:

1. Access the website: <http://www2.datasus.gov.br/DATASUS/index.php?area=0901> (Portal da Saúde);
2. Click the link "*SIHSUS*.". The website will load a new page;
3. Select the option "*Dados*" in "*Selecione uma ou mais modalidades de Arquivos para Download*" (select box);
4. Select the option "*RD - AIH Reduzida*" in "*Selecione um ou mais tipos de Arquivo*" (select box);
5. Select the year in "*Selecione o Ano*" (select box);
6. Select the Brazilian state in "*Selecione uma ou mais UF*" (select box);
7. Select the months in "*Selecione um ou mais Meses*" (select box);
8. Click on the button "*Enviar*". The website will re-load the page;
9. Click in the checkbox "*Marcar Todo*s";
10. Click on the button "*Baixar*". The website will load a new page;
11. Click in the generated link in the table column "*Arquivo*" (e.g. “Arq_xxxxxx.zip”). The browser will start to download the file;
12. After downloading the file, unzip it and use the program dbf2dbc.exe (Available for download at <http://www2.datasus.gov.br/DATASUS/index.php?area=060805&item=6>) to convert the .dbc files to .dbf. A dbf file can be opened in Microsoft Excel.

To get the CNES data the process is similar. The differences are in step 2 and 4:

1. Click the link "*CNES*". The website will load a new page;
2. Select the option "*Dados*" in "*Selecione uma ou mais modalidades de Arquivos para Download*" (select box);
3. Select the option "*ST - Estabelecimentos - A partir de Ago/2005*" in "*Selecione um ou mais tipos de Arquivo*" (select box) to get general Healthcare Facilities information, OR Select the option "*LT - Leitos - A partir de Out/2005*" in "*Selecione um ou mais tipos de Arquivo*" (select box) to get Healthcare Facilities bed information;
